# The Role of Maternal Depression in Racial Disparities and Birth Weight

**DOI:** 10.1007/s40615-025-02359-z

**Published:** 2025-03-04

**Authors:** Anna E. Eitel, Sara M. Witcraft, Bernadette Cortese, Ayesha C. Sujan, Courtney King, Constance Guille

**Affiliations:** 1 College of Medicine, Medical University of South Carolina, Charleston, SC USA; 2 Department of Psychiatry and Behavioral Sciences, Medical University of South Carolina, Charleston, SC USA; 3 Department of Anesthesiology, Perioperative and Pain Medicine, Stanford University School of Medicine, Stanford, CA USA; 4 Department of Obstetrics and Gynecology, Medical University of South Carolina, Charleston, SC USA

**Keywords:** Birth outcomes, Birth weight, Healthcare disparity, Maternal health, Perinatal care, Pregnancy

## Abstract

**Introduction:**

Pregnant people experiencing major depression during pregnancy are at increased risk for premature labor and infants with low birth weight, and there are significant racial disparities in these outcomes. Black women are at higher risk for having premature and low birth weight infants relative to their White counterparts. As such, we sought to examine the relationships between race, depression, and obstetric outcomes (low birth weight and prematurity) in both Black and White women with live births.

**Methods:**

This study included 185 pregnant women receiving behavioral health services within an Ob/Gyn clinic in an academic medical center in South Carolina. Main and interactive effects on birth weight and gestational age were evaluated with analysis of covariance controlling for maternal age.

**Results:**

The association between race and low birth weight was driven primarily by maternal depression. Infants of depressed Black women had significantly reduced birth weight relative to infants of depressed White women, but there was no evidence of racial disparities in birth weight among non-depressed Black women compared to non-depressed White women. Depression symptom severity was not associated with birth outcomes, and there was no effect of depression or race on prematurity.

**Conclusion:**

The occurrence of depression during pregnancy may in part account for racial disparities in infant birth weight. Interventions to reduce depression across birthing persons but especially among Black women may be a promising direction to address racial disparities in low birth weight.

## Introduction

Rates of premature or low birth weight infants are higher among Black women, compared to White women. A 2018 study, for example, reported the prevalence of preterm birth in Black women to be 12.2%, compared to 8.0% in White women [1]. Racial disparities in low birth weight are even more alarming. The rate of low birth weight has been reported at approximately twice as high for Black infants (11.36%), compared to White infants (5.21%) [2]. Prior research suggests that racial disparities in obstetric outcomes are potentially due to racial differences in socioeconomic status, age at pregnancy, marital status, quality of health care, genetic factors, neighborhood of residence, lifetime exposure to stress, and discrimination and racism [1, 3, 4]. Despite the identification of multiple risk factors that help explain the relationship between race and obstetric outcomes, none of these factors, alone or in combination, have been able to sufficiently explain this association [5], suggesting a more complex and dynamic association.

The biopsychosocial model was originally proposed as an alternative to the biomedical model in the 1970s and posits that health and illness result from a complex interaction between biological, psychological, and social/environmental factors [6]. Thus, a biopsychosocial conceptualization accounts for not only biological underpinnings, but also social determinants of health, environmental factors, and emotional and behavioral dimensions of health and illness. Few studies have applied the biopsychosocial model to perinatal or obstetric outcomes [7, 8], and none have examined racial disparities in perinatal health through a biopsychosocial lens. Using the biopsychosocial model as a framework may yield a more nuanced understanding of the relationship between race and obstetric outcomes.

There are numerous social and environmental factors that have been associated with increased risk for prematurity or low birth weight among Black women, including racism, discrimination, financial strain, parenting at a young age, lack of social support or health care, or living in an unsafe or impoverished neighborhood [9]. Compared to White women, Black women disproportionately experience stressful life events stemming from structural and social determinants of health (e.g., racism, poverty) as well as a greater number of adverse childhood events [10]. Adverse childhood events have been related to preterm birth, with Giurgescu and colleagues crediting a deduction in gestational weeks of 0.063 for each unit increase of maternal adverse childhood experience [11]. Further, the adversities of Black children are 33% higher than those of White children, and in a sample of Black women, 70% reported at least one adverse event during childhood [11]. Though these social and environmental factors alone do not yield poor obstetric outcomes [5], they provide both proximal and distal context for the disparities experienced by Black women.

In consideration of psychological factors, major depressive disorder (including peripartum depression) is strongly linked with a history of adverse life events and social constructs including race and socioeconomic status [12]. Additionally, risk factors that predispose Black women to have infants born early or with low birth weight (i.e., biological factors) mirror those for peripartum depression and include socioeconomic status, age at pregnancy, marital status/social support, genetics, community of residence, adverse life events, and discrimination [13]. Importantly, women experiencing major depression and psychological stress during pregnancy have an increased risk for premature or low birth weight infants [11, 14–17]. Despite the linkages between these constructs, studies have not examined the relationship between race, depression, stress, and obstetric outcomes [18]. Establishing an association between the risk of poor obstetric outcomes (biological), depression (psychological), and race (social) has important implications for developing interventions with a biopsychosocial framework to reduce racial disparities in poor obstetric outcomes. As such, the aim of this study was to examine the relationship between maternal depression and race and risk for prematurity or low birth weight.

## Methods

### Ethics Approval and Consent of Participants

This study was approved by the Institutional Review Board (IRB) at the Medical University of South Carolina (IRB protocol #00012855), and a waiver of written informed consent was granted by the IRB. This study was performed in accordance with the Declaration of Helsinki.

### Participants

Five hundred twenty patient charts were initially collected for analysis in the study. The inclusion criteria stipulated that participants must have attended an outpatient obstetrics and gynecology (Ob/Gyn) clinic within an academic medical center, been referred for behavioral health services, meet with a psychiatrist who determined DSM-IV/DSM-V diagnoses, and complete a routine battery of assessments for clinical care during their pregnancy. Additionally, participants must have had a live birth with accompanying infant data. Due to the IRB waiver of informed consent, all the women meeting these criteria were included in the study. The participants inclusion period spanned from July 2015 to June 2019.

### Measures

As part of routine mental health care, participants used REDCap (research electronic data captured), a secure online database, to complete standardized rating scales of current psychiatric symptoms and measures to gather demographic information (i.e., age, race, relationship status, annual income, years of education) via self-report. Participants were able to report their race and ethnicity with the following options: American Indian or Alaska Native, Asian, Native Hawaiian or Other Pacific Islander, Black or African American, White or Caucasian, and not Hispanic or Latino or Hispanic or Latino, respectively. Patients who identified with a Hispanic or Latino ethnicity were included within the White mother sample.

The 9-item Patient Health Questionnaire (PHQ-9) [15] is a commonly employed measure of depressive symptoms. For each of the 9 depressive symptoms, respondents indicate whether, during the previous 2 weeks, the symptom had bothered them “not at all,” “several days,” “more than half the days,” or “nearly every day.” Each item yields a score of 0 to 3, with the total score ranging from 0 to 27. Scores of 10 to 14, 15 to 19, and 20 or greater correspond to moderate, moderately severe, and severe depression, respectively.

Based on a clinical interview, psychiatrists entered in REDCap current and past DSM-IV/DSM-V diagnoses and treatment history. Electronic Health Records were reviewed, and obstetrical outcomes (e.g., birth weight and weeks’ gestation at delivery) were collected.

### Statistical Analysis

SPSS^©^ Version 24 was utilized for all analyses. Demographic and clinical characteristics were assessed with chi-square and independent *t*-tests. Main and interactive effects on birthweight and gestational age were evaluated with a two-way analysis of covariance (ANCOVA) controlling for the age of the mother.

## Results

### Participant Characteristics

One hundred and eighty-five pregnant women were included in the study. As a group, they had a mean age of 28.02 years (σ = 6.1; range = 13–43), were predominately White of non-Hispanic/Latina ethnic origin (67.6%), were mostly married or living with their partners (59.4%), and were diverse with respect to education level and socioeconomic status (Table 1). Ninety-five (51.4%) met DSM-IV/V criteria for mood disorder. Table 1 includes detailed characteristics of the sample separated by diagnosis. There were 33 (62.3%) Black women with and 20 (37.7%) without a diagnosis of depression and 62 (47.0%) White women with and 70 (53.0%) without a diagnosis of depression. Diagnostic groups did not differ with respect to race, education, socioeconomic status, and cigarette use (all *p*s > 0.1). While not clinically meaningful, the depressed group was statistically younger (27.1 vs. 29.1 years; *t*_185_ = 2.24, *p* = 0.03). As expected, the severity of depressive symptoms, measured by the PHQ-9 at mental health intake, was significantly greater in those diagnosed with mood disorder (*t*_185_ =  − 4.74, *p* < 0.001).

**Table 1:** Demographic and clinical characteristics of study participants (*N* = 185)

	Depressed^a^	Non-depressed	χ^2^ or *t*	*p*
	(*n* = 95)	(*n* = 90)		
Race, *n* (%) Black	33 (34.7)	20 (22.2)	3.54	0.06
Age in years (mean ± SD)	27.1 ± 6.0	29.1 ± 6.1	2.24	0.03
Education, *n* (%) college degree^b^	27 (31.4)^c^	34 (40.4)^d^	1.52	0.22
Income, *n* (%) < 50 k/yr^e^	57 (68.7)^f^	57 (67.1)^g^	0.05	0.82
Cigarette, *n* (%) smokers	29 (30.5)	32 (35.6)	0.52	0.467
Severity of depression (mean ± SD)^h^	13.6 ± 6.9	9.1 ± 5.9	− 4.74	< 0.001

^a^Current DSM-IV or DSM-V diagnosed mood disorder

^b^*n* = 170

^c^*n* = 86

^d^*n* = 84

^e^*n* = 168

^f^*n* = 83

^g^*n* = 85

^h^Patient Health Questionnaire 9 (PHQ-9) score

### Birth Weight

**Fig. 1:**
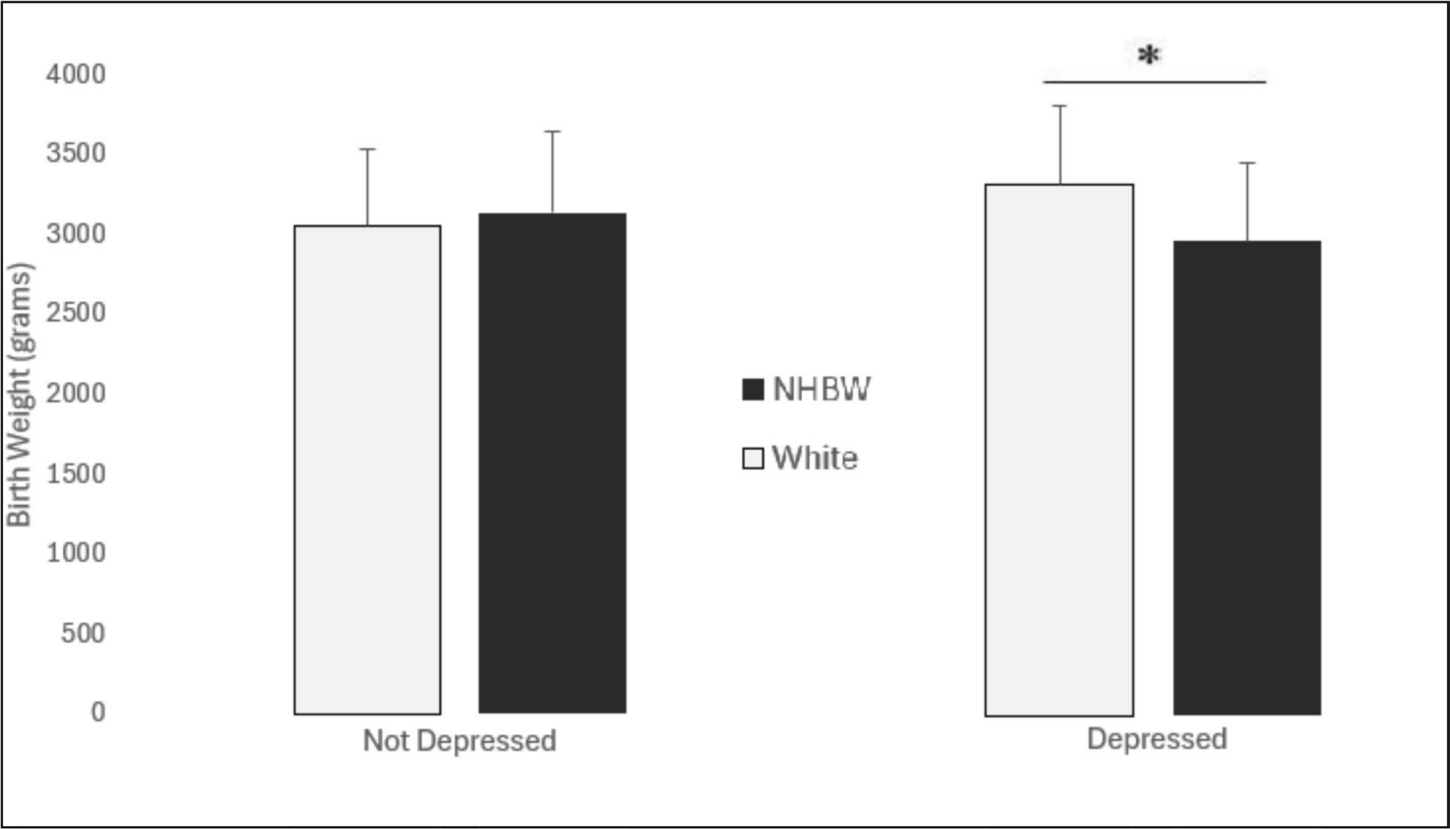
Infants of the depressed non-Hispanic black women (NHBW) were significantly reduced in birth weight compared to the infants of the depressed White women. In contrast, race was not a factor in the non-depressed group, as non-depressed NHBW and White women had infants with similar birth weights

ANCOVA, with age as a covariate, was used to evaluate associations between gestational depression, race, and infant birth weight. There was no significant effect of depression (*p* = 0.59) or race, collapsed across diagnosis (*p* = 0.15), on infant birth weight. However, a significant diagnosis by race interaction (*F*[1,851] = 7.74, *p* = 0.006) demonstrated that the association between race and reduced birth weight was associated with a major depressive disorder diagnosis. Infants of depressed Black women exhibited significantly reduced birth weight compared to infants of depressed White women (2953.8 ± 489.9 g vs. 3306.5 ± 491.7 g; *t*_95_ = 3.33, *p* = 0.0012; Fig. 1). In contrast, non-depressed Black women and non-depressed White women had infants with similar birth weights (3136.15 ± 511.3 g vs. 3052.2 ± 481.155 g; *t*_90_ = 0.67, *p* = 0.50; Fig. 1). Further analysis revealed that the severity of depression was unrelated to the effect on birth weight, as baseline PHQ-9 scores were similar between racial groups (Black women, 12.5 ± 7.1; White women, 10.8 ± 6.6; *p* = 0.3) and employing severity of depressive symptoms as a covariate in the analysis did not change the overall statistical findings.

### Gestational Age

ANCOVA, with age as a covariate, revealed that depression and race were not associated with gestational age at birth (*p* = 0.60 and 0.98, respectively), nor was there a significant interaction effect between depression diagnosis and race (*p* = 0.20). Unlike the results for birth weight, neither group (depressed or non-depressed), when assessed separately, showed any significant relationship between race and gestational age (*p* = 0.30 and 0.20, respectively).

## Discussion

This study examined the role of race and depression with respect to prematurity and low birth weight. In this study, racial disparities in birth weight appear to be driven primarily by a diagnosis of depression. In fact, no evidence of racial disparities in birth weight was found among non-depressed Black women compared to non-depressed White women. Interestingly, infants born to depressed White women exhibited the highest birth weights in the sample, which further underscores the racial disparities associated with maternal depression.

Although previous research has documented a link between prenatal depression and poor obstetric outcomes and shown racial disparities in poor obstetric outcomes, to our knowledge, this study is the first to demonstrate that maternal depression may contribute to racial disparities in obstetric outcomes [19–23]. The effect of depression on birth outcomes may be attributable to low socioeconomic status or psychosocial stress associated with lower income; however, it is also possible that depression on birth outcomes may be attributable to other racial/ethnic group differences that are associated with depression such as racism and discrimination [1, 3, 4, 24].

### Clinical Implications

Clinically, low birth weight is defined as an infant birth weight of less than 2500 g and is associated with various medical complications in both infancy and adulthood [25]. In the sample, non-Hispanic Black women exhibited the lowest average birth weight, with the standard deviation encompassing infants born below the 2500-g threshold. This finding emphasizes the clinical implications of racial disparities observed in depression and low birth weight, reinforcing the need for a deeper understanding of the biopsychosocial factors underlying this association.

Further, findings from the present study have important implications for developing interventions to reduce the risk of adverse birth outcomes, particularly for Black women. Interventions that lessen stress, anxiety, and depressed mood during pregnancy, such as supportive programs, relaxation techniques, and mindfulness, reduce the risk of preterm birth and low birth weight [26, 27], and cognitive behavioral therapy shows the most evidence for reducing depressive symptoms in Black pregnant individuals [28]. Additionally, since experiences of racial discrimination may increase the odds of depression [29], interventions for the prevention and treatment of depression in Black women should explicitly assess and address racism. Yet Black women are less likely to accept medication or psychotherapy for postpartum depression than White women, but more likely to accept spiritual counseling [30]. Treatment models for depression among Black women should use shared decision-making processes that empower the woman to dictate the course of treatment—whether pharmacotherapy, psychotherapy, both, or neither—and incorporate other supports including case management, spirituality, and family systems [31, 32]. This work underscores the need for better screening, identification of depression, and more available, accessible, and patient-informed treatment options for Black women.

### Research Implications

The findings demonstrate the need for more effective mental health interventions tailored to the unique experiences of Black women to mitigate racial disparities in birth outcomes. The development of these interventions needs to be informed by the input and perspectives of Black women, highlighting a critical area for future research. Further, additional studies are needed to explore potential mechanisms linking stressful events, depression, and adverse obstetric outcomes among Black women. This necessitates examining the interplay between inherent biological stress response systems and their interactions with social and psychological factors, thereby deepening the understanding of the biopsychosocial dynamics within this population.

The hypothalamic pituitary adrenal (HPA) axis is a biological system activated in response to stress and plays a critical role in stress regulation through the steroid hormone cortisol [33]. Cortisol exhibits a bimodal relationship with stress, wherein levels increase during acute stress and decrease during prolonged, chronic stress [33]. Previous literature has demonstrated that blunted cortisol levels during pregnancy are associated with higher perceived stress and Black race, likely secondary to the chronic stress induced by structural racism, discrimination, and social determinants of health [33]. Elevated cortisol levels can also negatively affect fetal development, leading to dysregulated organ development and premature labor-triggering stimuli [34]. Further, a positive correlation between cortisol levels and the presence of depression, a psychological factor, has been observed [35].

The interplay between biological, psychological, and social factors is further highlighted by the role of HPA axis dysregulation in the development of perinatal depression, prematurity, and low birth weight [13, 17, 36–39]. This connection between the three facets of the biopsychosocial model may help explain the relationship between race, stress, depression, and adverse obstetric outcomes. Moreover, previous literature has shown a higher risk of preterm birth in women with a more recent onset of depression compared to those who engaged with treatment for chronic depression [34]. Although the hypothesis remains untested, the finding suggests that treatment of depression could potentially interrupt a harmful inflammatory response [34, 40].

Recognizing and understanding the intricate interconnections between biological, psychological, and social factors within the broader context of the biopsychosocial model are vital for addressing racial disparities in birth outcomes and promoting maternal and fetal health. By integrating the understanding of psychological symptoms and their effects on molecular physiology, the treatment of maternal stress and depression provides a potential mechanism to attenuate and normalize the dysregulation of the HPA axis, particularly among populations disproportionately affected by chronic stress and health disparities. This approach may ultimately contribute to improved birth outcomes and reduced health inequities.

### Strengths and Limitations

Strengths of this study include a clinical sample of peripartum women with well-characterized mental health and substance use conditions and treatment information. Of note, because our analyses were conducted on a clinical sample, our comparison group included women with psychiatric disorders and, therefore, provided a more suitable match than had we used a non-clinical sample. Additionally, a strength of this study is the prospective data collection thereby limiting recall bias and its influence on all study measures. Further, self-report depressive symptoms and distress were completed online and likely reduced social desirability bias.

There are also limitations to the study. As this was an observational study, we could not determine causality. For example, while we observed that depression was related to lower birth weights among infants born to Black women, we cannot conclude that depression was the cause of the reduced birth weights. Also, the options of “Mixed” or “Other” were not provided for participants during the self-report of race, potentially influencing our results. Lastly, we did not collect data regarding the participants’ potential family history of mental illness, which is another significant cause of mental morbidity which is unable to be assessed in our study.

## Conclusion

In conclusion, this study provides novel data that contribute to a growing understanding of maternal depression and racial disparities in poor obstetric outcomes. These data delineate promising future directions to address racial disparities in low birth weight by enhancing engagement in treatment and improving treatment options for peripartum Black women.

## Data Availability

The data that support the findings of this study are available from the corresponding author, AE, upon reasonable request.
